# How to Publish: Guidance From GSA’s Journal Editors

**DOI:** 10.1093/geroni/igab046.758

**Published:** 2021-12-17

**Authors:** Suzanne Meeks

## Abstract

Each year he GSA publications team sponsors a symposium to assist authors who wish to publish in GSA’s high impact and influential journals. The first part of the session will include five brief presentations from the editors of The Gerontologist, Innovation and Aging, and the Journals of Gerontology Series A and B plus GSA’s managing editors. We will integrate practical tips with principles of publication ethics and scholarly integrity. The topics will be as follows: (1) Preparing your manuscript: strong and ethical scholarly writing for multidisciplinary audiences, (2) common problems that affect peer review, (3) addressing translational significance and fit to journal expectations, (4) transparency, documentation, and Open Science; and (5) working with Scholar One. Following these presentations, we will hold round table discussions with editors from the GSA journals portfolio. At these round tables, editors will answer questions related to the podium presentations and other questions specific to each journal. Intended audiences include emerging and international scholars, and authors interested in learning more about best practices and tips for getting their scholarly work published.

